# Postinduction Supportive Care of Pediatric Acute Myelocytic Leukemia: Should Patients be Kept in the Hospital?

**DOI:** 10.1155/2014/592379

**Published:** 2014-09-29

**Authors:** Susumu Inoue, Isra'a Khan, Rao Mushtaq, Dawn Carson, Elna Saah, Nkechi Onwuzurike

**Affiliations:** ^1^Hurley Children's Hospital and Hurley Medical Center, One Hurley Plaza, Flint, MI 48503, USA; ^2^Department of Pediatrics/Human Development, Michigan State University College of Human Medicine, One Hurley Plaza, Flint, MI 48503, USA

## Abstract

Children with AML become profoundly neutropenic while they undergo remission induction chemotherapy. It is unknown whether these children should be kept in the hospital while they are severely neutropenic to prevent life-threatening complications associated with neutropenia and reduce fatality. We at our institution routinely discharge patients after completing remission induction chemotherapy in the presence of profound neutropenia, unless it is clinically contraindicated. We reviewed all AML patients who were consecutively treated at our hospital from 1989 to 2011. Thirteen patients were electively discharged after completion of induction I chemotherapy. Of the 13, 4 died due to relapse or complications of stem cell transplants (not due to neutropenia related complications). Another eight are long term survivors. In this very small series, discharge from the hospital even though patients were severely neutropenic did not adversely affect the survival.

## 1. Introduction

Prognosis of childhood AML has improved partly due to improvement in the supportive care. Prophylactic antibiotics and antifungal agents during critical neutropenic periods have been documented to have contributed to this improvement [1–4]. Some investigators have recommended keeping patients in the hospital during severely neutropenic periods. Children's Oncology Group (COG) AML protocol, AML0531, for example, suggested continuation of hospital care until the ANC (absolute neutrophil count) reaches 200. Whether it is effective in increasing long term survival rate by keeping children in the hospital during the critical neutropenic period or not has not been conclusively answered. A COG study with an aim to address this question showed no difference in the rate of nonrelapse related mortality [1]. A few adult studies that examined mortality due to infection and/or hemorrhage managed as outpatients following induction or consolidation showed no increased mortality compared to those managed as inpatients, though readmission rates were high [5–7]. Our hospital does not have a policy in mandating to keep severely neutropenic patients hospitalized after the end of induction chemotherapy unless patients are febrile or meet any of the criteria described below. We have retrospectively reviewed our clinical records for all consecutively diagnosed AML patients at our hospital. At our hospital, patients were electively discharged once the induction chemotherapy is completed regardless of the absolute neutrophil count (ANC) unless patients are febrile or clinically ill. We sought to determine whether patients who were electively discharged at end of induction I chemotherapy had similar event-free survival rate (EFS) compared to the group wide survival rates of children treated with identical therapeutic protocols (POG or COG AML protocols).

## 2. Subjects

Subjects consisted of* all children consecutively diagnosed* to have AML except for FAB M3 (APL) between 1989 and 2011 at this institution. All children were treated with one of POG (Pediatric Oncology Group) or COG treatment protocols either on or off clinical trials. When no AML open protocols were available, we used the most recent closed phase III POG or COG protocols. There were 26 patients (13 males and 13 females) including 2 female patients with Down syndrome. In one of them, the AML followed Down syndrome-associated neonatal myeloproliferative disorder. Of the total of 26 patients, thirteen were electively discharged after 1st remission induction chemotherapy since they had no complications that would have necessitated continuous hospitalizations. The other 13 continued hospitalizations due to intercurrent complications such as fever, skin infection, and abdominal pain. Thus the subjects for the purpose to answer the question were the 13 patients who were electively discharged.

## 3. Supportive Care

All patients were started on trimethoprim-sulfa for* Pneumocystis jiroveci* prophylaxis during the 1st induction period and placed on oral fluconazole for fungus prophylaxis. No broad spectrum antibiotics were prescribed prophylactically. When the ANC fell below 200, hard foods and roughage (e.g., potato chips) and raw fruits and vegetables intake were strongly discouraged to minimize injury to the mucous membrane. Platelet transfusions were empirically given when platelet counts dropped below 20,000/*μ*L. RBC (leukocyte reduced) transfusions were given at Hb of 7-8 g/dL. G-CSF (Neupogen) use was limited to culture positive life threatening infections with severe neutropenia. At the completion of induction I chemotherapy, patients were discharged regardless of the neutrophil counts or platelet counts if patients appeared clinically stable and well without fever and had no severe nausea/vomiting, diarrhea, abdominal pain, or local wounds such as lacerations or perianal fissures. CBC and leukocyte differentials were daily performed. Screening coagulation profiles or any routine imaging studies such as a routine chest X-ray were not performed. They were done when clinically indicated. Social and nonmedical factors such as an overcrowded home, the caretaker feeling totally overwhelmed by the medical circumstances, or living a long distance from the hospital were factors that prevented the attending physician from discharging the patient, even though chemotherapy was completed. The parents or guardians were instructed to call the on-call hematologist whenever the body temperature measured 100.3°F, 38°C, or above. A febrile (fever ≥ 38.3°C) neutropenic patient (ANC < 500) was generally immediately hospitalized to the pediatric floor without an evaluation in our emergency department during nights and weekends. During the business hours these patients were first evaluated at hematology/oncology clinic, unless the patient was deemed unstable. Accessing central venous catheters at ED was strongly discouraged. Febrile neutropenic patients upon admission were empirically placed on ceftazidime prior to the year 2000 and subsequently on meropenem. Routine empirical use of vancomycin was avoided. If the patients remained neutropenic (ANC < 300) and febrile for longer than 3 days without positive blood cultures, amphotericin B or liposomal amphotericin was empirically added. Oral mouthwash such as chlorhexidine oropharyngeal or another mouthwash was routinely prescribed. Patients were generally followed twice a week in the clinic during the extreme neutropenic and thrombocytopenic period.

All patients who were treated on POG protocols were referred to an outside stem cell transplant center. If the patient was found to have an HLA matched sibling, they were transplanted in remission. Patients who were treated on COG AAML0531 were also transplanted if they had HLA matched siblings. The exceptions were those whose leukemic cells showed 8 : 21 translocation, in which case continuation of chemotherapy was selected after discussion with the parents. One patient with M4e with inv 16 received allogeneic marrow transplant. All relapsed patients received reinduction chemotherapy and were given stem cell transplants either with unrelated matched donors, cord blood, or haplomatched siblings. Rare refractory patients who failed to enter remission were give stem cell transplants in relapse.

## 4. Results

The total number of subjects consecutively diagnosed to have AML during this period was 26 excluding patients with APL (M3) (13 male and 13 female). This included 2 Down syndrome patients. The age ranged from 9 months to 14 years. FAB classification of the diagnoses was M0, 2 patients, M1, 3 patients, M2, 6 patients, M4, 7 patients, M5a and M5b, 1 patient each, M6, 2 patients, and M7, 4 patients (2 of them had Down syndrome). The follow-up period ranged from 20 days to 24 years with median of 4.5 yrs. Fourteen patients were treated with POG 9421 induction regimen and 8 patients with COG AAML 0531 induction regimen. Two patients were treated with POG 8821, and one patient each was on COG AAML0431 and COG AAML 03P1, respectively. Four patients had the typical *t* (8 : 21) translocation, and one patient had M4e with inversion of 16. All of these patients are alive and in remission. One patient with Down syndrome (M7) developed respiratory failure during the 1st induction course and was placed on ECMO but died. Of the 26 patients, 13 patients (50%) were electively discharged upon completion of induction I chemotherapy in spite of profound pancytopenia. The remaining 13 patients were kept in the hospital due to the following reasons: febrile neutropenia (4 patients), bacterial sepsis (4 patients), typhlitis (2 patients), enterocolitis (1 patient), and respiratory failure needing ECMO (1 patient). One patient was electively kept in the hospital due to severe neutropenia due to a long distance from home, though there was no complication that would necessitate hospitalization.

**Figure 1:**
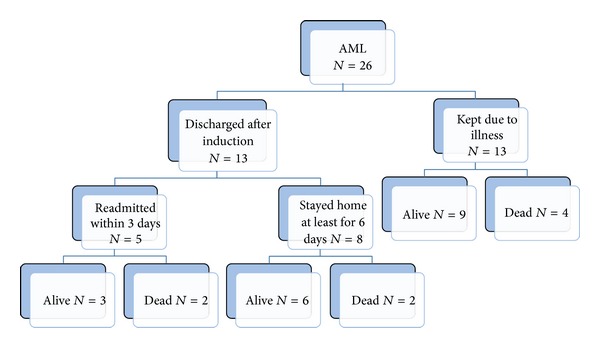
Ultimate outcome of patients who were electively discharged after induction I and those who were kept in the hospital.

The outcome of electively discharged patients is shown in Figure 1. Of the 13 patients electively discharged, all patients were eventually hospitalized again due to complications. Reasons for readmission and the day of admission following discharge are shown in Table 1. Five patients were readmitted within 3 days after the discharge. Three of the five were readmitted due to febrile neutropenia, one patient due to* α hemolytic strep* sepsis, and one 8-month-old patient due to dehydration. None of these 5 died of immediate complications of chemotherapy or leukemia. The remaining 8 patients stayed home for at least 6 days after discharge. The 2 patients of this group eventually died of relapse. The reasons for late readmission for the 8 patients were listed in the table. Two patients of the 8 eventually died of relapse or complications of stem cell transplants. Thus none of the patients who were discharged early had fatal complications following the induction I chemotherapy. Eleven patients received bone marrow or cord blood transplants, eight of them from matched sibling donor, and the remainder from MUD or cord blood.

The survival rate of the total AML group was 69% (18/26) which is better than the overall group wide results of POG9421 (overall EFS at 3 years 41.2%) (COG meeting report, Fall 2001) or COG AAML0531 (DFS at 3 years 61% with gemtuzumab ozogamicin) (COG meeting report, Fall 2013).

## 5. Discussion

Historically some of the intensive AML chemotherapy regimens carried very high nonrelapse related mortality [8, 9]. Modification of chemotherapeutic regimens and better supportive care decreased this therapy related mortality rate in recent years [1, 2, 8, 10]. A recent article on the adult AML therapy stated “it is standard practice that newly diagnosed adult AML patients having received initial induction chemotherapy remain hospitalized “preemptively” until blood count recovery” [11]. This may be true for adult patients, but there are significant differences between adults and children [12] and this practice has not been uniformly observed in pediatric institutions both in and outside the United States [13]. A majority (55%) of COG participating institutions have had a policy or guidelines to keep patients hospitalized during severely neutropenic periods [13], and 21% of COG institutions routinely discharged AML patients before bone marrow recovery. In contrast, fifty percent of BFM participating institutions routinely discharged patients before bone marrow recovery. Interestingly, a study performed by the Pediatric Oncology Group found that mandatory hospitalization during profound neutropenia did not reduce infection rate or significantly reduce nonrelapse-related mortality [1]. Our findings here, though the number is small, are consistent with this COG group wide finding. Of the 13 patients electively discharged, 9 patients are long term survivors, and 4 patients who died all succumbed to relapse of the disease or complications of marrow transplants after relapse. Of the other 13 patients who remained in the hospital due to illnesses and complications, the same number of 9 patients is alive, and four died. Thus, elective discharge upon completion of 1st induction chemotherapy before marrow recovery did not affect the long term prognosis of patients, though the number is very small.

There are some unique local factors that need to be considered before the result can be generalized. First, this is a community hospital, and oncology patients are housed in the general pediatric unit. The unit lacks any laminar air flow room, and thus risk of nosocomial infection is high. This is one of the factors why we do not keep neutropenic patients in the hospital unless there are overriding concerns. There are only two pediatric hematologists/oncologists so that the physicians have personal knowledge of virtually all patients who are treated. Further one of the two is always on call and is able to respond to any questions or concerns parents have at any time of the day. In addition most patients are within 2 hours of driving distance from the hospital. Therefore we are able to start systemic antibiotics promptly after a patient is noted to be febrile. These factors may have contributed to the good results.

For a child, quality of life is unquestionably better when he or she is at home no matter how sick the patient is. Appetite and energy level usually increase once a child is discharged to home compared to when a child is in the hospital, particularly for toddlers and infants. Even though many patients who were discharged after completion of induction I chemotherapy were rehospitalized, eight of 13 patients were able to stay home for 6 days or longer.

Limitations of this observation are many. The sample size is extremely small, and thus the outcomes described could be by chance alone. This is a single institution observation, and many local factors including human factors undoubtedly influenced the outcome, and thus the conclusion cannot be generalizable. It is a retrospective nonrandomized observational study with all of their limitations.

Nonetheless, we are reassured by this retrospective review of the fact that discharging patients before marrow recovery is not harming patient in the long run. Thus we feel it reasonable to continue this practice in the absence of published or unpublished contradictory data.

## Figures and Tables

**Table 1:** Details of subsequent hospitalizations on patients who were electively discharged after induction I chemotherapy.

Patient number	Age in year	Day of admission after discharge	Reasons for readmission
6	7	11	Febrile neutropenia
7	14	2	Febrile neutropenia
8	4	8	Alpha hemolytic strep sepsis
9	9 months	2	Dehydration
10	4	8	Foot cellulitis
12	11.5	10	Perianal cellulitis
13	13	6	Febrile neutropenia
14	11	12	Fever and herpes stomatitis
15	3	1	Alpha hemolytic strep sepsis
17	5	1	Febrile neutropenia
19	4	7	Febrile neutropenia
20	2	1	Febrile neutropenia
25	22 months	10	Febrile neutropenia
